# Enhancing semantic interoperability in precision medicine: converting OMOP CDM to Beacon v2 in the Spanish IMPaCT-Data project

**DOI:** 10.1186/s12911-026-03649-0

**Published:** 2026-07-08

**Authors:** Manuel Rueda, Juan Manuel Ramírez-Anguita, Victoria López-Sánchez, Sergi Aguiló-Castillo, Maria Eugenia Gas López, Alberto Labarga, Miguel-Ángel Mayer, Javier Ripoll Esteve, Ivo G. Gut

**Affiliations:** 1 Centro Nacional de Análisis Genómico (CNAG), Baldiri Reixac 4, Barcelona, 08028 Spain; 2 Universitat de Barcelona (UB), Barcelona, Spain; 3 Research Programme on Biomedical Informatics, Hospital del Mar Medical Research Institute and Universitat Pompeu Fabra, Barcelona, Spain; 4 Hospital del Mar, Barcelona, Spain; 5 Instituto de Investigación Sanitaria La Fe (IIS La Fe), Valencia, 46026 Spain; 6 Barcelona Supercomputing Center (BSC), Barcelona, 08028 Spain; 7 Spanish National Bioinformatics Institute (INB/ELIXIR-ES), Barcelona, Spain

**Keywords:** OMOP Common Data Model (OMOP CDM), GA4GH Beacon v2, Semantic interoperability, Precision medicine, Clinical data integration, Health data interoperability, Electronic health records, FAIR data, Open-source software, Clinical data mapping

## Abstract

**Objective:**

To evaluate a file-based approach for converting OMOP CDM data into GA4GH Beacon v2 format and outline an alternative on-the-fly architecture for real-time access, with the aim of enhancing semantic interoperability within Spain’s IMPaCT-Data program for personalized medicine.

**Materials and methods:**

We used the Convert-Pheno tool to transform OMOP CDM exports into Beacon v2 format using a file-based approach. In addition, we describe an architecture that connects PostgreSQL-based OMOP CDM instances directly to the Beacon v2 API, enabling real-time data access without intermediary text files.

**Results:**

We successfully converted OMOP CDM datasets from three research centers (CNAG, IIS La Fe, and HMar) to Beacon v2 format with high data completeness (≥ 99.9% across most domains).

**Discussion and conclusion:**

This study evaluates a file-based approach and describes an alternative on-the-fly architecture for integrating OMOP CDM data with Beacon v2. These approaches provide practical options for integrating OMOP CDM data with Beacon v2 in different deployment settings.

**Supplementary Information:**

The online version contains supplementary material available at https://doi.org/10.1186/s12911-026-03649-0.

## Introduction

Semantic interoperability is a key requirement for precision medicine, particularly when integrating genomic and clinical data at scale [1, 2]. In this regard, the OMOP Common Data Model (CDM) is a widely adopted framework for harmonizing clinical data, powered by the OHDSI standardized vocabulary [3]. However, its design lacks native support for genomic data, a critical component in personalized medicine.

To address this limitation, previous work has proposed extending OMOP CDM to incorporate genomic data, such as the Genomic Common Data Model (G-CDM) [4]. However, these efforts have seen limited adoption and ongoing development, and OMOP CDM still lacks standardized, widely supported native genomic capabilities. These efforts align with parallel initiatives such as the Global Alliance for Genomics and Health (GA4GH), which introduced Beacon v2 [5] and Phenopacket v2 [6] standards for federated genomic and phenotypic data sharing [7, 8]. Rather than replacing OMOP CDM, the goal of GA4GH standards is to provide structured, interoperable data schemas for seamless integration across systems.

The Spanish National Infrastructure for Precision Medicine (IMPaCT) program aims to embed precision medicine into the Spanish National Health System, ensuring equitable access to cutting-edge genomic healthcare (https://impact.isciii.es/*).* Within this, the IMPaCT-Data initiative consolidates diverse datasets, leveraging Beacon v2 to enable robust genomic and phenotypic data discovery (https://impact-data.bsc.es/en/about/impact*).*

**Fig. 1:**
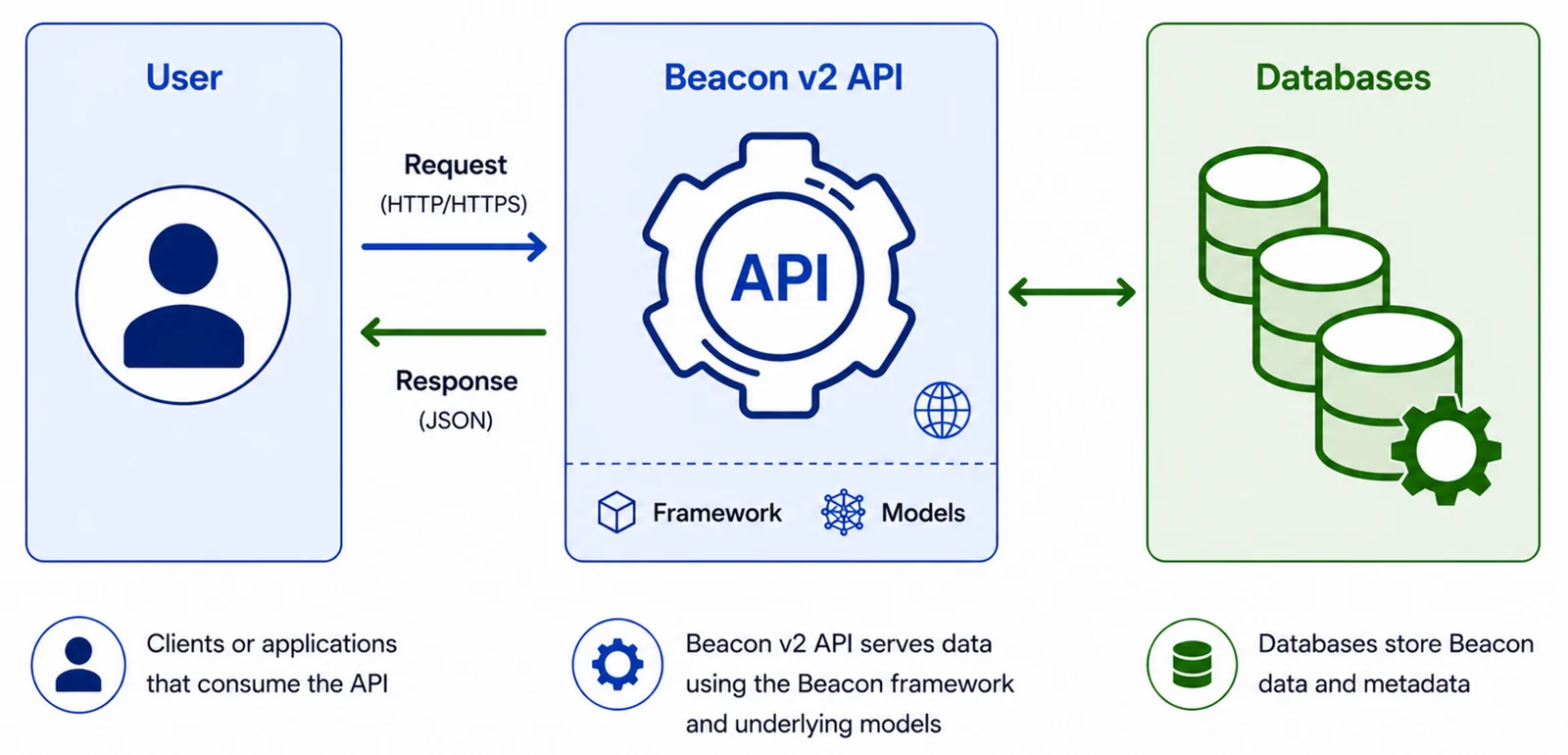
Schematic overview of the Beacon v2 API. User queries are processed through the API framework and models, which retrieve and return data from underlying data sources. In this study, these correspond to non-relational databases containing Beacon v2–formatted collections or OMOP CDM instances, depending on the implementation approach

This work focuses on the design, implementation, and evaluation of interoperable healthcare information system architectures that align OMOP CDM with the GA4GH Beacon v2 specification. Within the IMPaCT program, we evaluate a file-based approach for the conversion of OMOP CDM data to Beacon v2 across multiple clinical datasets, and describe an alternative on-the-fly architecture that enables direct, real-time access to OMOP CDM data through the Beacon v2 API (Fig. 1 provides a schematic overview of the Beacon v2 API and its query–response structure). By addressing real-world data transformation challenges, this study provides practical insights to support federated health data interoperability.

## Materials and methods

The goal of this study is to enable researchers to query OMOP CDM patient data via the Beacon v2 API (see Fig. 1), particularly for identifying individuals with specific characteristics or phenotypes. To achieve this, we implemented a file-based approach and developed an on-the-fly approach: (i) a file-based conversion, leveraging preformatted JSON data stored in a non-relational database like MongoDB, and, (ii) an on-the-fly conversion, dynamically converting data from SQL-based relational databases (e.g., PostgreSQL) in real-time. These methods address different needs, such as efficient pre-processed data retrieval and real-time data access.

### Datasets

Three OMOP CDM datasets were used to evaluate the file-based conversion approach: (i) the synthetic EUNOMIA dataset, used as a controlled validation dataset because its synthetic nature allows comparison between input OMOP CDM records and generated Beacon v2 objects under reproducible conditions; (ii) a real-world COVID-19 clinical dataset from IIS La Fe, used to evaluate the conversion in a medium-scale clinical setting; and (iii) the IMASIS-2 dataset from Hospital del Mar, used to evaluate the approach in a large-scale real-world clinical dataset derived from routine hospital information systems. These datasets differ in size, origin, and complexity, allowing evaluation across synthetic, medium-scale clinical, and large-scale institutional scenarios. OMOP CDM version 5.4 was used in all implementations. Detailed conversion outcomes, including input record counts, generated Beacon v2 objects, and completeness metrics, are reported in the Results section and Supporting Tables.

### File-based conversion (Convert-Pheno)

OMOP CDM databases often store millions of rows of patient data, making complex, multi-table queries computationally intensive. To address this, we pre-formatted OMOP data into the Beacon v2 data exchange format, the Beacon Friendly Format (BFF), which supports efficient data discovery and interoperability [9].

The process begins by exporting PostgreSQL data as text files (.SQL or .CSV). These files are then transformed into JSON using Convert-Pheno [10], a tool developed under the 3TR project (https://3tr-imi.eu/*)* to convert clinical and phenotypic data into the Beacon v2 schema. The conversion preserves all OMOP CDM information relevant to the Beacon v2 *individuals* entity. Fields without direct equivalents are retained under the *info* term to ensure lossless transformation and standards compliance (see Supporting Text 1). The conversion process is typically performed once, with updates applied as new data becomes available. All conversions were performed using the Convert-Pheno command-line interface. For CSV inputs, the command ‘convert-pheno -iomop *.csv -obff individuals.json’ was used. For large SQL dump files, streaming mode was enabled using ‘convert-pheno -iomop dump.sql -obff individuals.json -stream -max-lines-sql 999999999’ to reduce memory usage during processing. A tutorial for this file-based approach is available on a Google Colab notebook (https://github.com/CNAG-Biomedical-Informatics/omop-cdm-2-beacon-v2).

We applied a multi-level validation strategy to assess correctness and completeness. Source datasets conform to the OMOP CDM 5.4 schema, and all Convert-Pheno outputs conform to the Beacon v2 schema, as compliance is enforced during transformation and verified using the ‘bff-tools validate’ command from the Beacon v2 Reference Implementation (B2RI) [9]. Completeness was assessed by comparing the number of OMOP CDM records (rows on each table) with the number of generated Beacon v2 objects (per term), where matching counts indicate successful mapping. Additional details on mapping, validation procedures and completeness calculations are provided in the Supplementary Material.

**Fig. 2:**
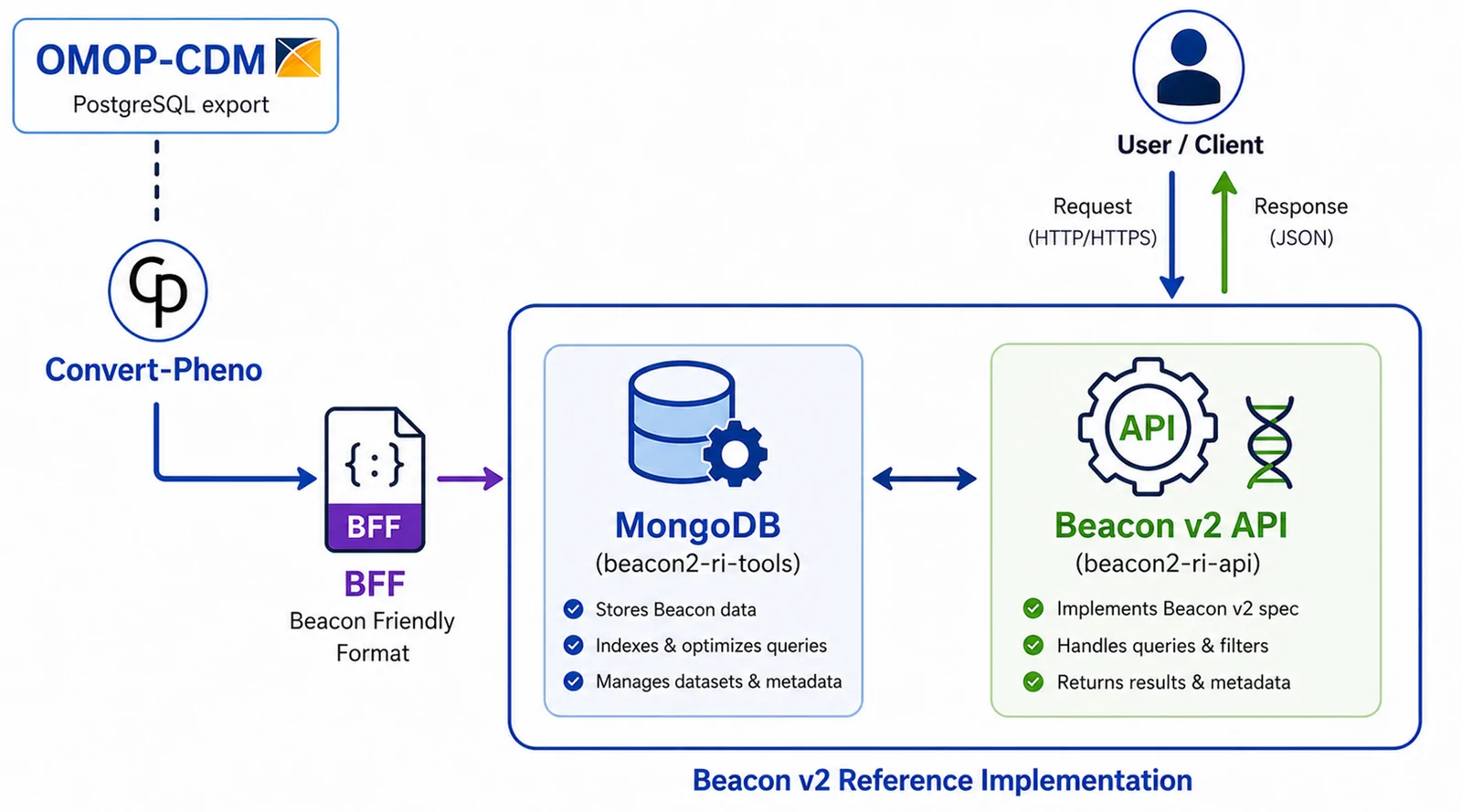
File-based OMOP CDM to Beacon v2 conversion workflow. OMOP CDM data are exported from PostgreSQL as SQL or CSV files and converted with Convert-Pheno into Beacon Friendly Format (BFF) JSON. The resulting (schema-validated) BFF collections can be ingested into a MongoDB backend and exposed through a Beacon v2 API implementation for standardized query and response

Once generated, the BFF JSON files can be loaded into a non-relational database, such as MongoDB. These collections are readily accessible through the B2RI [9] or other Beacon v2 APIs based on non-relational databases (see Supporting Text 1). The end-to-end file-based conversion workflow, from OMOP CDM extraction to Beacon v2-compatible JSON generation and database integration, is illustrated in Fig. 2.

### ‘On-the-fly’ conversion (Beacon v2 RI OMOP extension, ongoing development)

**Fig. 3:**
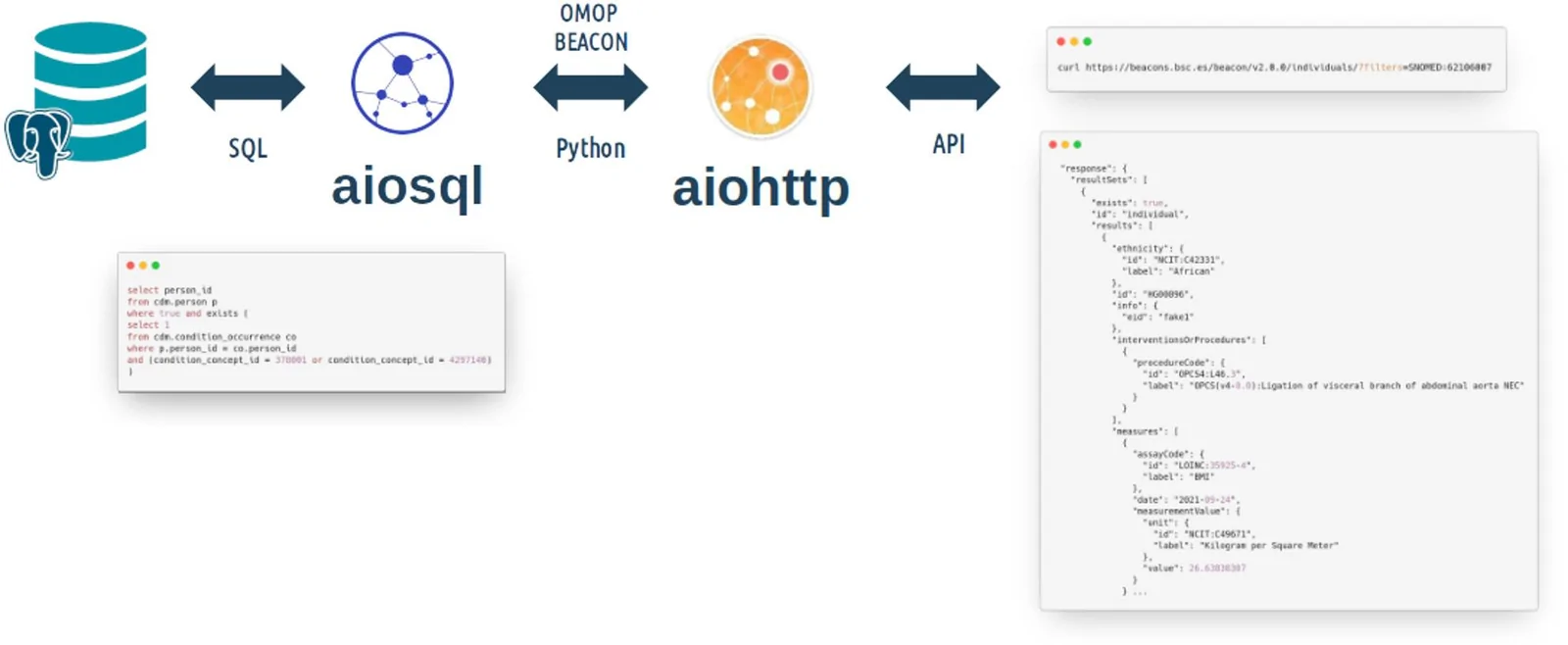
Current implementation of the Beacon v2 RI OMOP extension

**Fig. 4:**
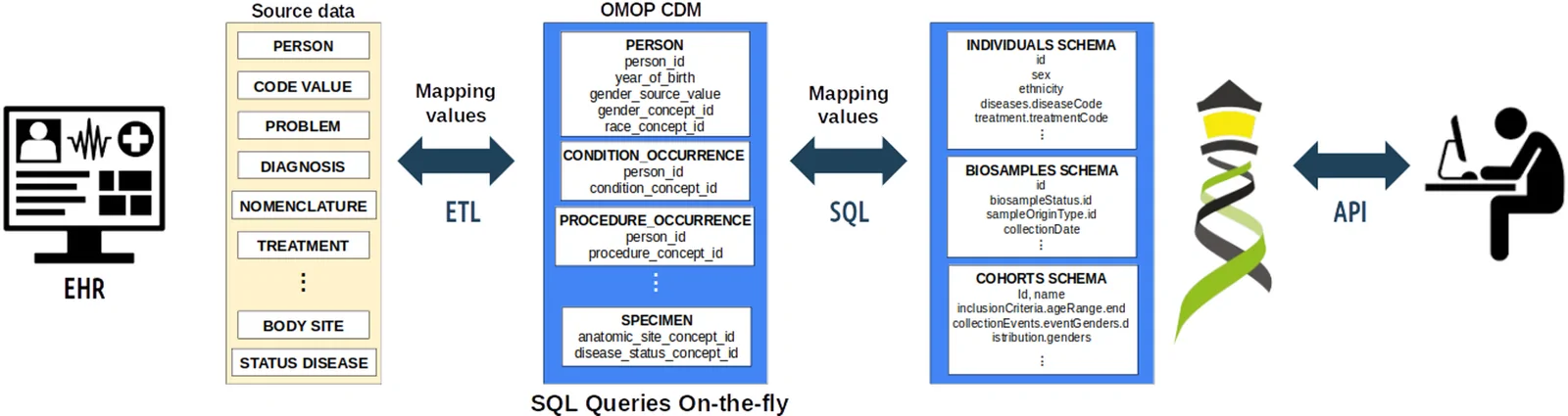
Schematic representation of the Beacon v2 RI OMOP extension

An alternative method involves directly linking the Beacon v2 API with the OMOP CDM database (see Figs. 3 and 4), a technique implemented by members of the Barcelona Supercomputing Center (BSC) as an extension of the B2RI (https://gitlab.bsc.es/impact-data/impd-beacon_omopcdm*)* [11, 12].

This ‘on-the-fly’ approach involves connecting the Beacon v2 API directly to the OMOP CDM database using SQL queries (see Fig. 4). The system translates the Beacon API requests into SQL queries that retrieve the necessary data directly from the database and transforms the data to JSON. This approach is designed to enable an up-to-date granular view of the data and eliminate the need for the intermediate data exchange files, periodic transformations and the maintenance of the MongoDB replica. This architecture is currently under active development and is described here to provide context for alternative deployment strategies; it has not yet been systematically evaluated in this study.

## Results

We evaluated the system across three real-world healthcare information system deployments as part of Spain’s IMPaCT-Data project (GdT05-03), converting three independent OMOP CDM instances to the Beacon v2 format. We used diverse clinical research databases, including a small dataset from the Centro Nacional de Análisis Genómico (CNAG) in Barcelona, a medium-scale dataset from the Health Research Institute Hospital La Fe (IIS La Fe) in Valencia, and a large dataset from Hospital del Mar (HMar) in Barcelona (see full details in Supporting Table 3).

### File-based conversion at CNAG (with synthetic data)

At CNAG, we evaluated the file-based approach using the synthetic EUNOMIA dataset (https://ohdsi.github.io/Eunomia/*)*, which includes records for 2,694 patients across multiple tables. This dataset was selected because its synthetic nature provides full control over both input and expected output, enabling reliable validation of the conversion process by comparing OMOP records with the generated BFF under controlled conditions. Data were loaded into PostgreSQL using R and exported as an .SQL file for transformation (see Table 1 and Supporting Table ST2 for details). Scripts are available upon request.

Table 1 summarizes the completeness of converting EUNOMIA table records to the Beacon v2 *individuals* entity. VISIT_OCCURRENCE values are excluded because visits may span multiple terms per individual (stored under the “_visit” property). Additionally, handling longitudinal data is beyond the scope of the Beacon v2 specification.

**Table 1:** Completeness on the conversion from OMOP CDM tables to Beacon v2 objects for the EUNOMIA synthetic dataset

OMOP tables	BFF term	Rows in CSV	Objects in BFF	Completeness (%)
Concept	-	444	-	-
Condition_occurrence	diseases	65,332	65,332	100
Drug_exposure	treatment	67,707	67,707	100
Measurement	measures	44,053	44,053	100
Observation	phenotypicFeatures	1,477	1,477	100
Person	id	2,694	2,694	100
Procedure_occurrence	interventionsOrProcedures	37,409	37,409	100
Visit_occurrence	_visit	1,037	-	-

### File-based conversion at IIS La Fe

The conversion process from an OMOP CDM instance to the *individuals* entity of the Beacon v2 Models was executed using real data from the Big Data Platform of the IIS La Fe. Specifically, a retrospective study was conducted using pseudo-anonymised clinical information collected from patients (both survivors and non-survivors) infected with COVID-19 between December 31, 2021 and February 28, 2022 with a confirmed diagnosis of COVID-19 via RT-qPCR.

Clinical information was extracted from various sources, including pharmaceutical prescriptions, symptomatology, clinical observations, hematology, biochemical and blood gas parameters, and hospital and primary care resources, all mapped to OMOP CDM. The resulting COVID-19 dataset encompassed records for 18,544 patients across several tables: CONCEPT, CONDITION_OCCURRENCE, DRUG_EXPOSURE, MEASUREMENT, OBSERVATION, PERSON, PROCEDURE_OCCURRENCE, and VISIT_OCCURRENCE. Each aforementioned table was exported from SQL databases to 8 CSV files. The Table 2 illustrates the result of the transformation of COVID-19 records into the *individuals* entity.

**Table 2:** Completeness on the conversion from OMOP CDM tables to Beacon v2 objects for the COVID-19 dataset

OMOP tables	BFF term	Rows in CSV	Objects in BFF	Completeness (%)
Concept	-	4,317,890		
Condition_occurrence	diseases	205,374	205,357	99.99
Drug_exposure	treatment	159,837	159,833	100
Measurement	measures	2,610,570	2,610,411	99.99
Observation	phenotypicFeatures	58,025	58,025	100
Person	id	18,544	18,543	99.99
Procedure_occurrence	interventionsOrProcedures	64,914	64,908	99.99
Visit_occurrence	_visit	137,103	-	-

### File-based conversion at HMar

The data conversion process begins with the IMASIS information system [13], the EHR system of Parc Salut Mar Barcelona, a comprehensive healthcare services organization. Currently, this information system includes and shares the clinical data of two general hospitals, one mental health care centre, one social-healthcare centre and five emergency room settings, which are offering different services in the Barcelona city area (Spain). IMASIS includes clinical information from around 1 million patients who have used the services of this healthcare system since 1990 and from different settings such as admissions, outpatients, emergency rooms, and major ambulatory surgery. The diagnoses are coded using The International Classification of Diseases ICD-9-CM [14] and ICD-10-CM [15]. IMASIS-2 is the anonymized relational database of IMASIS being the data source used for mapping to OMOP including additional sources of information such as the Tumours Registry.

As shown in Table 3, after the conversion, the completeness of the mappings approaches 100% across most domains, with minor discrepancies in PERSON and MEASUREMENT tables.

**Table 3:** Completeness on the conversion from OMOP CDM tables to Beacon v2 objects for the IMASIS-2 dataset

OMOP tables	BFF term	Rows in CSV	Objects in BFF	Completeness (%)
Concept	-	6,871,345	-	-
Condition_occurrence	diseases	7,837,107	7,837,107	100
Drug_exposure	treatment	73,400,506	73,400,506	100
Measurement	measures	248,897,994	248,885,056	99.99
Observation	phenotypicFeatures	1,672,053	1,672,053	100
Person	id	1,033,805	1,033,623	99.98
Procedure_occurrence	interventionsOrProcedures	1,815,641	1,815,641	100
Visit_occurrence**	_visit	16,270,148	-	-

**Table VISIT_OCCURRENCE was excluded from the calculation

## Discussion

This study evaluates the conversion of OMOP CDM data to Beacon v2 in real-world settings, complementing prior work on integrating OMOP with GA4GH standards like Phenopackets v2 [16–18]. The following sections outline the advantages of the two conversion approaches—file-based and on-the-fly—in different operational contexts (see also Table 4).

### When to use file-based conversion

The file-based approach is well-suited for centers leveraging non-relational databases, such as MongoDB, to manage genomic data derived from VCFs [19] alongside clinical data. It is particularly effective for projects consolidating data from multiple sources (e.g., REDCap, CDISC, OMOP CDM, CSV) into centralized storage, enabling standardized data sharing through a Beacon v2 API. Preformatted data enable fast API response times, making it ideal for workflows where data transformation occurs once data collection is complete. This scenario is typical of data submissions to repositories such as EGA [20] and dbGAP [21], which require the coordinated processing of both genomic and clinical data. However, this method relies on periodic exports to incorporate newly generated data, which may limit its suitability in highly dynamic environments. Despite this, the approach remains computationally efficient, compatible with standard server environments, and secure when deployed in controlled settings that safeguard data sensitivity and anonymity.

### When to use ‘On-the-fly’ Conversion

The on-the-fly conversion approach is suitable when access to up-to-date OMOP CDM data is required (e.g., updates to OMOP CDM are frequent). This approach is currently under active development and has not yet undergone systematic benchmarking; therefore, no quantitative performance results are reported here. It eliminates the need for periodic data transformation and avoids maintaining duplicate clinical data, thereby reducing the demand for additional computational resources. However, it requires a well-indexed and optimized database to support complex SQL queries efficiently and it is not ideal for scenarios requiring a unified backend with genomic data stored in a non-relational database (e.g., ingested BFF from processed VCFs). Within the IMPaCT project, this implementation is being developed in the context of the UNICAS pilot to support a federated pediatric network for rare diseases. The Beacon v2 RI OMOP extension is also being used for the COVID-NL dataset in the By-Covid project (https://by-covid.org/) and is planned for deployment in the DATOS-CAT project [22], which will provide access to the Spanish GCAT cohort data (http://www.gcatbiobank.org/).

**Table 4:** Comparison of file-based and ‘on-the-fly’ OMOP CDM to Beacon v2 conversion approaches

	File-Based Conversion	Direct SQL Query
Data Freshness	Data are static and requires manual updates	Real-time data access, ensuring the latest information is always available
Integration with Genomic Data	Unified Backend: Uses MongoDB to centralize clinical and genomic data (from VCFs).	Database Duplicity: Separate databases for genomic data (MongoDB) and clinical data (SQL-based)
Performance	Fast response with pre-formatted data	Fast response with properly indexed OMOP database
Implementation Complexity	Requires software installation and a secure environment to process data	Simplified setup by avoiding intermediate steps, directly querying the database
Resource Utilization	Requires storage proportional to data size. Data must be ingested and indexed into a non-relational database	Efficient use of storage as data remains in the database
Scalability	Scalable with pre-formatted (JSON Schema validated) data	Dynamically scalable with efficient SQL queries and robust and mature SQL technologies
Flexibility	Limited to Beacon v2 data schema	Highly flexible with dynamic queries
Maintenance	May require periodic updates	Lower maintenance with automatic data freshness
Data Security	Controlled data dumps disconnect Beacon v2 API requests from OMOP instances, adding a layer of security. Text files still require secure handling.	Enhanced security by keeping data within the secured database environment
Data Reusability and FAIRification**	Converted data can be directly deposited into repositories such as the EGA [20] or stored in data warehouses from EU-funded projects. Data are immediately available for analytics (e.g. [23])	Directly relies on open source OMOP tools like Atlas or HADES for flexible, FAIR-aligned data reuse and analysis

** Findable, Accessible, Interoperable, Reusable

### Limitations

The proposed approaches inherit limitations from the underlying standards and system architectures. Beacon v2 is optimized for federated data discovery rather than longitudinal clinical analysis and therefore does not fully capture visit-level temporal trajectories present in OMOP CDM. In addition, data completeness and semantic consistency depend on the quality of local OMOP implementations and vocabulary mappings. The choice between file-based and on-the-fly conversion involves trade-offs between data freshness, performance, and operational complexity across institutional settings. In addition, the on-the-fly approach has not yet undergone systematic benchmarking, and future work will focus on its quantitative evaluation.

Finally, exposing OMOP CDM data through a Beacon v2 interface introduces privacy and governance considerations that depend on the specific deployment context. Beacon v2 supports different response granularities, including Boolean, count/aggregate, and record-level responses, and the associated disclosure risks vary accordingly. Although Boolean or aggregate responses limit direct exposure of patient-level data, repeated or highly specific queries may still create inference risks, including membership inference, cohort differencing, or potential identification of individuals with rare clinical or genomic features. These risks have been previously described in the context of genomic data-sharing beacons [24, 25]. The present work focuses on the transformation of OMOP CDM data into a Beacon-compatible format and does not evaluate Beacon deployment, access-control, or query-governance configurations. These aspects should therefore be considered separately by each implementing institution, according to the response granularity exposed, the sensitivity of the underlying data, and the applicable data-access governance framework [26].

## Conclusion

This study describes two alternative approaches for transforming OMOP CDM data into the Beacon v2 schema: a file-based method for efficient preprocessed data retrieval and an on-the-fly conversion for real-time access. By offering practical guidance, we aim to support similar initiatives and encourage the broader adoption of Beacon v2.

These methodologies aim to improve the interoperability and accessibility of clinical and genomic data, fostering collaboration and supporting precision medicine infrastructures to enhance patient care.

## Supplementary Information

Below is the link to the electronic supplementary material.

Supplementary Material 1

## Data Availability

The synthetic EUNOMIA dataset used for evaluation in this study is publicly available from the OHDSI project (https://ohdsi.github.io/Eunomia/). A PostgreSQL export of the EUNOMIA dataset, used as input for the OMOP CDM to Beacon v2 conversion, is provided through the Convert-Pheno repository (https://github.com/CNAG-Biomedical-Informatics/convert-pheno). The complete data transformation workflow, including scripts and step-by-step instructions to reproduce the conversion using this dataset, is openly accessible via a Google Colab notebook available in the companion repository (https://github.com/CNAG-Biomedical-Informatics/omop-cdm-2-beacon-v2). Clinical OMOP CDM data from participating IMPaCT-Data institutions contain sensitive patient information and are subject to local data protection and governance regulations; therefore, these datasets are not publicly available.

## Abbreviations

API: Application Programming Interface
B2RI: Beacon v2 Reference Implementation
BFF: Beacon Friendly Format
CDM: Common Data Model
CSV: Comma-Separated Values
EGA: European Genome-phenome Archive
EHDEN: European Health Data Evidence Network
EHR: Electronic Health Record
GA4GH: Global Alliance for Genomics and Health
OHDSI: Observational Health Data Sciences and Informatics
OMOP: Observational Medical Outcomes Partnership
RT-qPCR: Reverse Transcription quantitative Polymerase Chain Reaction
VCF: Variant Call Format
