# Supplementary material for: Enhancing semantic interoperability in precision medicine: converting OMOP CDM to Beacon v2 in the Spanish IMPaCT-Data project
Source: BMC Med Inform Decis Mak. 2026 Jul 8;26:389. doi: 10.1186/s12911-026-03649-0 (PMC13637149; doi:10.1186/s12911-026-03649-0)
Supplement: Supplementary file 1 [file 12911_2026_3649_MOESM1_ESM.pdf]

Supplementary Data

Enhancing Semantic Interoperability in Precision Medicine: Converting OMOP CDM to Beacon v2 in the Spanish IMPaCT-Data Project

Manuel Rueda[1, 2, †, *], Juan Manuel Ramírez-Anguita[3, †], Victoria López-Sánchez[5, †], Sergi Aguiló-Castillo[6, 7], Maria Eugenia Gas López[5], Alberto Labarga[6,7], Miguel-Ángel Mayer[3,4], Javier Ripoll Esteve[5] and Ivo G. Gut[1,2]

[1] Centro Nacional de Análisis Genómico (CNAG), Baldiri Reixac 4, 08028 Barcelona, Spain

[2] Universitat de Barcelona (UB), Barcelona, Spain

[3] Research Programme on Biomedical Informatics, Hospital del Mar Medical Research Institute and Universitat Pompeu Fabra, Barcelona, Spain.

[4] Hospital del Mar, Barcelona, Spain.

[5] Instituto de Investigación Sanitaria La Fe (IIS La Fe), 46026, Valencia, Spain.

[6] Barcelona Supercomputing Center (BSC), 08028 Barcelona, Spain.

[7] Spanish National Bioinformatics Institute (INB/ELIXIR-ES), Spain.

[†] These authors contributed equally to this work

Sup. Data Contents:

BFF integration with the Beacon v2 API STxt1
Mapping between OMOP CDM and Beacon v2 schemas .... STxt2 and ST1
Comparative Performance Metrics for Data Conversion Across Different Centers........ ST2
References

Supporting Text 1: BFF integration with the Beacon v2 API

Phenotypic and clinical data from the OMOP CDM v5.4 schema were integrated into Beacon v2, a standard for genomic and phenotypic data discovery. Specifically, the integration targeted the *individuals* entity of the Beacon v2 Models (https://docs.genomebeacons.org/), which includes terms such as *diseases*, *ethnicity*, *exposures*, *geographicOrigin*, *id*, *info*, *interventionsOrProcedures*, *karyotypicSex*, *measures*, *pedigrees*, *phenotypicFeatures*, *sex*, and *treatments*. These elements capture both patient and healthy control data, supporting a wide range of use cases in precision medicine.

The workflow involves creating a JSON output file (e.g., 'individuals.json') using the Convert-Pheno tool [1]. Conversion was performed using the command-line interface (CLI) with the default command: 'convert-pheno -iomop file.sql -obff individuals.json -max-lines-sql 999999'. By default, Convert-Pheno operates in 'no-stream' mode, loading all patient data from SQL tables into RAM. Rows are transposed and merged into single patient objects (see Supporting Text 2 and Table ST1). For memory-limited environments, the 'stream' mode processes OMOP tables row by row, using the term *id* as primary key to avoid loading all data into memory. During conversion, 'concept_id' values in the CONCEPT table remain unchanged, except for the *sex* term, which is mapped to NCIt terminology. Necessary 'concept_id' entries are included to maintain schema consistency.

This 'individuals.json' file is ingested into a MongoDB collection, with MongoDB serving as the database backend for the Beacon v2 Reference Implementation API (*beacon2-ri-*

*ap*i) [2]. Figure 2 in the manuscript illustrates the end-to-end process, from OMOP CDM data extraction to Beacon v2-compatible JSON generation and MongoDB integration.

In this setup, the MongoDB instance is deployed in a dedicated Docker container for scalability and isolation. The *beacon2-ri-api* was initially chosen for its compatibility with BFF-based MongoDB workflows, with deployment documentation available at https://github.com/EGA-archive/beacon2-ri-api. Alternative APIs, such as BSC's Java-based implementation (https://github.com/ga4gh-beacon/java-beacon-v2) [3] or the newest *beacon2-pi-api* (https://github.com/EGA-archive/beacon2-pi-api), can also be used.

Supporting Text 2 and Table 1: Mapping between OMOP CDM and Beacon v2 schemas

The OMOP Common Data Model (CDM) v5.4 employs a relational SQL schema (https://ohdsi.github.io/CommonDataModel/cdm54.html), whereas Beacon v2 adopts a hierarchical JSON schema (https://docs.genomebeacons.org/schemas-md/individuals_defaultSchema). Bridging these formats requires converting a relational schema into a hierarchical representation while preserving data consistency.

To address this, schema mappings were developed during the creation of the Convert-Pheno tool [1]. These mappings align the relevant OMOP CDM tables with corresponding elements in the Beacon v2 *individuals* schema. During development, outputs were iteratively validated using the 'bff-tools validate' command from the B2RI [2], ensuring that all generated JSON files conform to the Beacon v2 schema, as compliance with required structure and data types is enforced during transformation. This process refined the OMOP-to-Beacon mappings and ensured structural consistency prior to database ingestion.

Validation was further supported by complementary approaches. For the synthetic EUNOMIA dataset, where expected outputs are known, programmatic validation was performed under controlled conditions. In addition, domain experts conducted targeted manual validation of representative mappings to assess semantic consistency. For the large-scale IMASIS dataset, Convert-Pheno's mapping was iteratively refined in collaboration with data owners, enabling the identification and resolution of dataset-specific edge cases not captured in smaller or synthetic datasets.

The latest OMOP CDM to Beacon v2 mappings and step-by-step instructions are available at https://cnag-biomedical-informatics.github.io/convert-pheno/omop2bff/.

Key Considerations in Mapping:

1. Lossless Data Transition: To ensure that no data are lost, original OMOP variables are retained within the Beacon v2 *info* term. Columns without a direct mapping are stored under “info” or “_ + variable_name”, preserving the original OMOP context. This facilitates cross-checking by domain experts and allows original OMOP CDM values to be directly queried through the Beacon v2 API when needed.
2. Custom Selection of Exposures: For the Beacon v2 *exposures* term, a manual selection of commonly used ‘concept_id’ values was derived from the OMOP CONCEPT table (see https://github.com/CNAG-Biomedical-Informatics/convert-pheno/blob/main/share/db/concepts_candidates_2_exposure.csv).

Process Overview:

The mapping converts OMOP CDM records from relational fact tables into nested objects within the Beacon v2 *individuals* entity. Patient-level data from the PERSON table define the Beacon v2 individual identifier and demographic attributes, while clinical event tables (CONDITION_OCCURRENCE, DRUG_EXPOSURE, MEASUREMENT, PROCEDURE_OCCURRENCE, and OBSERVATION) are transformed into structured arrays within each individual, corresponding to *diseases*, *treatments*, *measures*, *interventionsOrProcedures*, and *phenotypicFeatures*.

OMOP vocabulary fields ('concept_id', 'concept_name', 'vocabulary_id', and 'concept_code') are used to populate ontology-style id and label attributes in Beacon. Supporting Table 1 provides concrete examples of these transformations.

Definition of Completeness:

Completeness was defined as the proportion of OMOP CDM records successfully represented in the corresponding Beacon v2 entities:

$$Completeness = \frac{N_{Beacon\ objects}}{N_{OMOP\ records}}\ x\ 100$$

where $N_{OMOP\ records}$ is the number of rows in each OMOP CDM table and $N_{Beacon\ objects}$ is the number of generated objects in the corresponding Beacon v2 term. Matching counts indicate successful mapping, reflecting record-level completeness, as only mapped OMOP records generate Beacon v2 objects.

Supporting Table 1: Example of Beacon v2 *individuals* entity terms mapped from clinical events in OMOP CDM.

<table>
<tr><th>Beacon Term (Individuals)</th><th>BFF (output)</th><th>OMOP "Fact table"</th><th colspan="2">OMOP mapping based on</th></tr>
<tr><td rowspan="3">ethnicity</td><td rowspan="3">"ethnicity": {
"id": "NCIT:C41261",
"label": "White"
}</td><td rowspan="3">person</td><td>concept_id</td><td>8527</td></tr>
<tr><td>domain_id</td><td>race</td></tr>
<tr><td>concept_name</td><td>White</td></tr>
<tr><td>id</td><td>"id": "1"</td><td>person</td><td>person_id</td><td>1</td></tr>
<tr><td rowspan="2">sex</td><td rowspan="2">"sex": {
"id": "NCIT:C20197",
"label": "Male"
}</td><td rowspan="2">person</td><td>gender_concept_id</td><td>8507</td></tr>
<tr><td>domain_id</td><td>gender</td></tr>
<tr><td>info</td><td>"info": {
"PERSON": {
"OMOP_columns": {
(…) }
},</td><td>person</td><td></td><td></td></tr>
<tr><td rowspan="7">diseases</td><td rowspan="7">"diseases": [
{
"_info": {
"CONDITION_OCCURRENCE": {
"OMOP_columns": {
(…) }
},
"ageOfOnset": {
"age": {
"iso8601duration": "32Y"
}
},
"diseaseCode": {
"id": "SNOMED:444470001",
"label": "Injury of anterior cruciate ligament"
}</td><td rowspan="7">condition_occurrence</td><td>condition_concept_id</td><td>40479768</td></tr>
<tr><td>domain_id</td><td>condition</td></tr>
<tr><td>concept_name</td><td>Injury of anterior cruciate ligament</td></tr>
<tr><td>condition_start_date</td><td>‘ ’</td></tr>
<tr><td>birth_date time (person)</td><td>1949-01-27 00:00:00.000</td></tr>
<tr><td>vocabulary_id</td><td>SNOMED</td></tr>
<tr><td>concept_code</td><td>444470001</td></tr>
</table>

**interventionsOrProcedures**	**"interventionsOrProcedures"**: [<br>{<br>"_info": {<br>"PROCEDURE_OCCURRENCE": {<br>"OMOP_columns": {<br>(...) }<br>},<br>"ageAtProcedure": {<br>"age": {<br>"iso8601duration":"32Y"<br>}<br>},<br>"dateOfProcedure": "1981-08-17",<br>"procedureCode": {<br>"id": "SNOMED:699253003",<br>"label": "Surgical manipulation of joint of knee"<br>}	procedure_occurrence	procedure_concept_id	44783196
			domain_id	procedure
			concept_name	Surgical manipulation of joint of knee"
			procedure_start_date	‘’
			birth_date time (person)	1949-01-27 00:00:00.000
			vocabulary_id	SNOMED
			concept_code	699253003
**treatments**	**"treatments"**: [<br>{<br>"_info": {<br>"DRUG_EXPOSURE": {<br>"OMOP_columns": {<br>(…) }<br>},<br>"ageAtOnset": {<br>"age": {<br>"iso8601duration": "36Y"<br>}<br>},<br>"doseIntervals": [],<br>"routeOfAdministration": {<br>"id": "NCIT:N0000",<br>"label": ""<br>},<br>"treatmentCode": {<br>"id": "RxNorm:140587",<br>"label": "celecoxib"<br>}	drug_exposure	drug_concept_id	1118084
			domain_id	drug
			concept_name	celecoxib
			drug_exposure_start_date	‘’
			birth_date time (person)	1949-01-27 00:00:00.000
			vocabulary_id	RxNorm
			concept_code	140587

Supporting Table 2: Comparative Performance Metrics for Data Conversion Across Different Centers

	**CNAG**	**IIS La Fe**	**HMar**
**Data Type / Name**	Synthetic / EUNOMIA	Real / COVID-19	Real / IMASIS 2
**# Patients**	2,694	18,554	1,033,805
**# Records**	~220 K	~8 M	~350 M
**Timing / Hardware****	~5 sec / Intel® Xeon® W-1350P CPU @ 4.00GHz with 32GB RAM	< 4 min / Intel® Core™ i9-13900K with 125 GB RAM	7.1 hours / Intel® Xeon® CPU W-2265 @ 3.50GHz with 188 GB RAM
**Input Type / Size**	PostgreSQL export / 33 MB (7MB gz)	CSVs / 944 MB (127 MB .tar.gz)	PostgreSQL export / 56 GB (8.1 GB gz). The dump creation took ~6 minutes.
**Output (JSON array) Size**	2,694 objects / 354 MB (19 MB gz)	18,544 objects/ 6 GB (225 MB gz)	333.6 million objects / 1.2TB (19 GB gz)
**Mode / RAM Usage**	No-stream / ~500 MB	No-stream / ~15 GB	Stream / ~8 GB

** 1 core. Further information on system requirements can be found at the online documentation (https://cnag-biomedical-informatics.github.io/convert-pheno/omop-cdm/#omop-as-input).

References:

1. Rueda M, Leist IC, Gut IG. Convert-Pheno: A software toolkit for the interconversion of standard data models for phenotypic data. J Biomed Inform. 2024;149:104558. https://doi.org/10.1016/j.jbi.2023.104558.

2. Rueda M, Ariosa R, Moldes M, Rambla J. Beacon v2 Reference Implementation: a toolkit to enable federated sharing of genomic and phenotypic data. Bioinforma Oxf Engl. 2022;38:4656–7. https://doi.org/10.1093/bioinformatics/btac568.

3. Repchevsky D, Capella-Gutierrez S, Gelpí JL. Open source Java implementation of the Beacon v2 API. 2022. https://doi.org/10.7490/F1000RESEARCH.1118980.1.
